# Advanced machine learning in action: identification of intracranial hemorrhage on computed tomography scans of the head with clinical workflow integration

**DOI:** 10.1038/s41746-017-0015-z

**Published:** 2018-04-04

**Authors:** Mohammad R. Arbabshirani, Brandon K. Fornwalt, Gino J. Mongelluzzo, Jonathan D. Suever, Brandon D. Geise, Aalpen A. Patel, Gregory J. Moore

**Affiliations:** 1 Geisinger, Department of Radiology, 100 N. Academy Avenue, Danville, PA 17822-2007 USA; 2 Geisinger, Department of Imaging Science and Innovation, 100 N. Academy Avenue, Danville, PA 17822-4400 USA

**Keywords:** Brain imaging, Computed tomography

## Abstract

Intracranial hemorrhage (ICH) requires prompt diagnosis to optimize patient outcomes. We hypothesized that machine learning algorithms could automatically analyze computed tomography (CT) of the head, prioritize radiology worklists and reduce time to diagnosis of ICH. 46,583 head CTs (~2 million images) acquired from 2007–2017 were collected from several facilities across Geisinger. A deep convolutional neural network was trained on 37,074 studies and subsequently evaluated on 9499 unseen studies. The predictive model was implemented prospectively for 3 months to re-prioritize “routine” head CT studies as “stat” on realtime radiology worklists if an ICH was detected. Time to diagnosis was compared between the re-prioritized “stat” and “routine” studies. A neuroradiologist blinded to the study reviewed false positive studies to determine whether the dictating radiologist overlooked ICH. The model achieved an area under the ROC curve of 0.846 (0.837–0.856). During implementation, 94 of 347 “routine” studies were re-prioritized to “stat”, and 60/94 had ICH identified by the radiologist. Five new cases of ICH were identified, and median time to diagnosis was significantly reduced (*p* < 0.0001) from 512 to 19 min. In particular, one outpatient with vague symptoms on anti-coagulation was found to have an ICH which was treated promptly with reversal of anticoagulation, resulting in a good clinical outcome. Of the 34 false positives, the blinded over-reader identified four probable ICH cases overlooked in original interpretation. In conclusion, an artificial intelligence algorithm can prioritize radiology worklists to reduce time to diagnosis of new outpatient ICH by 96% and may also identify subtle ICH overlooked by radiologists. This demonstrates the positive impact of advanced machine learning in radiology workflow optimization.

## Abstract (longsummary)

A computer program that automatically analyzes brain images from patients undergoing CT scans of the head can reliably flag those with signs of hemorrhage. A team of researchers from Geisinger in Danville, Pennsylvania, USA, trained and tested a machine-learning algorithm using 46,583 computed tomography imaging studies of the head. Subsequently, they implemented the model into routine care for 3 months to help prioritize radiology worklists. Of 347 routine cases, the computer identified 94 as having an intracranial hemorrhage, two-thirds of which were confirmed by a radiologist, including five among patients who had a new diagnosis of a brain bleed. The algorithm reduced the average time in which a radiologist diagnosed these patients from around 8.5 h to just 19 min, demonstrating the positive impact of incorporating artificial intelligence into radiology workflow.

## Introduction

Intracranial hemorrhage (ICH) is a critical condition accounting for about 2 million strokes worldwide.^1^ Hemorrhages can occur both within the brain parenchyma (intra-axial) or within the cranial vault but external to the brain parenchyma (extra-axial). Both intra-axial and extra-axial hemorrhage have significant clinical burden. For example, intra-axial hemorrhage affects approximately 40,000 to 67,000 patients per year in the United States^2,3^ with a 30-day mortality rate of 47 percent.^4^ Moreover, 46% of survivors of subarachnoid hemorrhage (a type of extra-axial hemorrhage) endure permanent cognitive impairment.^5,6^ Hospital admissions of ICH have dramatically increased in the past decade probably due to increased life expectancy and poor blood pressure control.^7,8^ Importantly, early diagnosis of ICH is of critical clinical importance since nearly half of the resulting mortality occurs in the first 24 h,^9^ and earlier treatment likely improves outcomes.^10^ Computed tomography (CT) of the head is the most widely used tool for diagnosing acute ICH, and the timing of diagnosis, therefore, depends on how quickly a head CT is both completed and subsequently interpreted by a clinician.

The interpretation time of radiological studies is highly dependent on the priority assigned to the exam by the ordering physician (for example “stat” vs “routine”) and by patient status (inpatient vs. outpatient). Stat studies are typically interpreted within an hour (at our institution) while routine outpatient studies can take much longer based on the available radiology workforce. Therefore, detection of ICH in routine studies (especially those imaged in an outpatient setting) may be significantly delayed. ICH does occur in the outpatient setting, albeit with a lesser frequency than the inpatient or emergency department setting. For example, elderly outpatients on anticoagulation therapy experience higher risk of ICH.^11^ Importantly, initial symptoms may be vague, prompting a non-emergent, routine head CT.

Automatic triage of imaging studies using computer algorithms has the potential to detect ICH earlier, ultimately leading to improved clinical outcomes. Such a quality improvement tool could be used to automatically manage the priority for interpretation of imaging studies with presumed ICH and help optimize radiology workflow. Machine learning and computer vision are among a suite of techniques for teaching computers to learn and detect patterns.

In particular, deep learning (a class of machine learning algorithms suitable for training large multi-layer neural networks) has been leveraged for a variety of automated classification tasks such as natural language processing, speech recognition and object detection.^12^ There has been growing interest in “augmented” diagnostic vision with machine learning recently in the medical field.^13^ For example, a recent paper showed that diabetic retinopathy can be accurately detected from retinal photographs using deep learning.^14^ Additional published applications include detection and diagnosis of skin cancer,^15^ pulmonary nodules^16^ and cerebral microhemorrhage.^17^ Despite these studies demonstrating the promise of machine learning to positively impact the field of diagnostic medicine and radiology, clinical implementation of deep learning thus far is rare.

The purpose of this study was twofold. The first goal was to develop a predictive deep learning model capable of detecting ICH based on a large clinical database of head CT studies. Second, we aimed to implement and test the predictive model in real time as a radiology workflow optimization tool. We hypothesized that this implementation as a quality improvement tool would lead to a significant reduction in the average interpretation time of head CTs showing ICH without significantly adding to the burden of “stat” studies on the radiology worklists. To our knowledge, no study has reported using a large cross-sectional imaging database and advanced computer vision techniques for detection of critical radiological findings in a quality improvement setting. Moreover, clinical implementation of an automatic deep learning based radiology quality improvement tool and subsequent workflow optimization has, to our knowledge, never been performed.

## Results

**Fig. 1:**
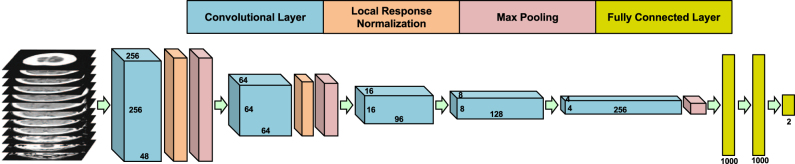
Proposed CNN architecture for ICH detection. This architecture has five convolutional and two fully connected layers

**Fig. 2:**
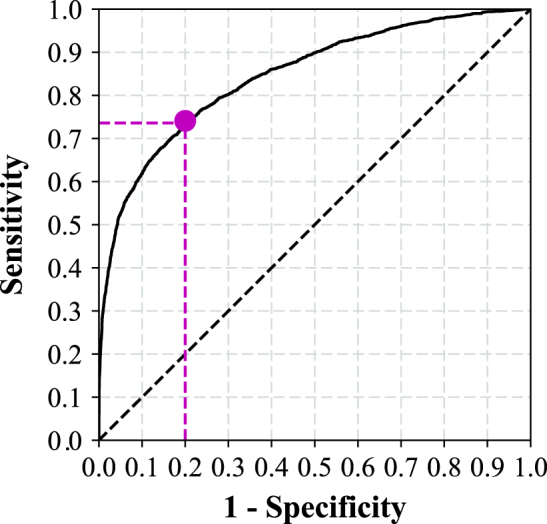
ROC curve of the ICH detection algorithm on the testing dataset. The magenta circle illustrates the operating point. AUC of the ROC is 0.846

**Fig. 3:**
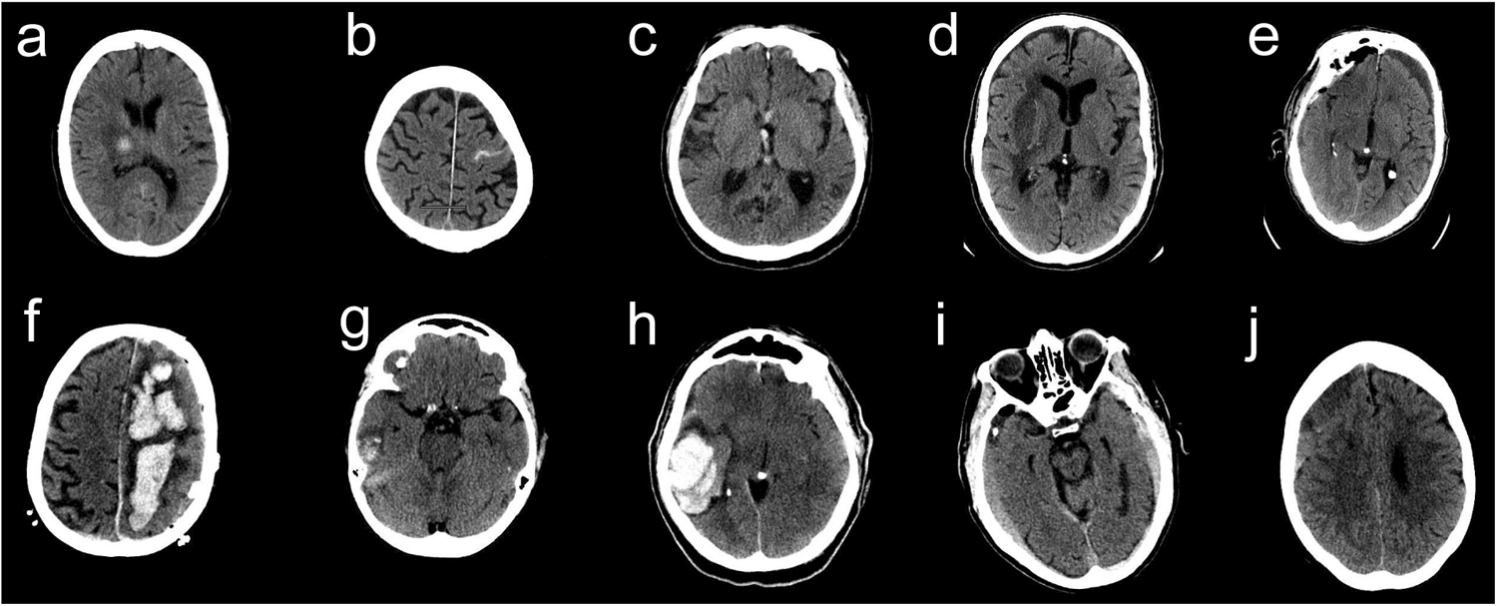
Examples of true positive ICH cases detected by the algorithm. **a** Outpatient with right thalamic hemorrhage. **b** Outpatient with subarachnoid hemorrhage in the left frontal convexity. **c** Inpatient with acute intraventricular hemorrhage within the third ventricle. **d** Outpatient with right basal ganglia/external capsular hemorrhage. **e** Inpatient with chronic subdural hemorrhage. **f** Outpatient with large left frontoparietal intraparenchymal hemorrhage. **g** Inpatient with intraparenchymal hemorrhagic contusion in the right temporal lobe. **h** Inpatient with large intraparenchymal hematoma. **i** Outpatient with bilateral subdural hemorrhages. **j** Outpatient with subdural hemorrhage

The fully 3-dimensional deep learning architecture developed and used in this study to classify the presence or absence of ICH within head CT studies (analyzed as a complete 3-dimensional study, not as individual images) is illustrated in Fig. 1 and described in detail in the methods. After training the algorithm on 37,084 head CT studies, the receiver operating characteristic (ROC) curve was generated using the 9499 unseen testing datasets (Fig. 2). The area under the curve (AUC) of the model was 0.846 (95% CI: 0.837–0.856) for predicting the presence of absence of ICH. A false positive rate of 0.2 was chosen for the operating point (magenta circle on Fig. 2), where the overall specificity and sensitivity were 0.800 (95% CI: 0.790–0.809) and 0.730 (95% CI: 0.713–0.748), respectively. The algorithm detected a variety of ICH cases despite the heterogeneity in the clinically-acquired data. Figure 3 illustrates examples of the correctly detected ICH cases (true positives). Examples of false positives can be found in the supplementary material (Figure S1).

### Clinical implementation results

**Fig. 4:**
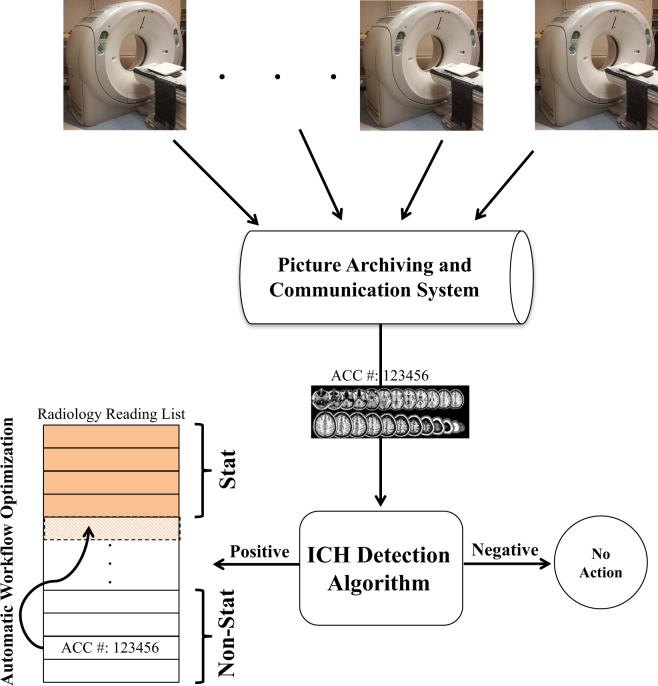
Clinical implementation of the ICH detection algorithm as a radiology workflow optimization tool

The clinical implementation phase utilized the algorithm to reprioritize radiology worklists (Fig. 4). This portion of the study ran from 6 January 2017 to 24 March 2017, during which time 347 “routine” head CT studies were processed using the algorithm with an accuracy of 84% (CI: 78–87%), sensitivity of 70% (CI:58–78%) and specificity of 87% (CI:82–91%). Of the 347 “routine” studies, 94 cases were upgraded to “stat” on the worklist (26%) and, of these, 60 were dictated as having an ICH present by the interpreting radiologist (resulting in a positive predictive value of 60/94 = 64%). Importantly, of these 94 cases, five new ICH cases from outpatients were detected (5%). The complete confusion matrix is provided in the supplemental material (table S1). The average processing time for running the ICH detection algorithm on a given study was 2.3 sec. The studies prioritized as “stat” had a median time to clinical interpretation of 19 min (IQR: 22 min), which was significantly lower (*p* < 0.0001) than the median interpretation time for the “routine” studies (512 min, IQR: 1551 min). The clinical details of two selected cases are reviewed below.

#### Case #1

**Fig. 5:**
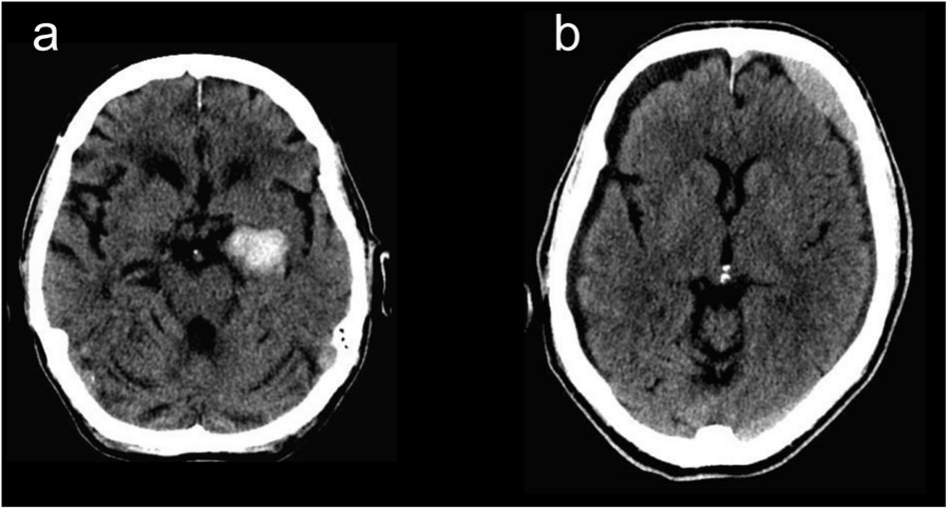
Representative head CT image slices associated with case #1 (**a**) and case #2 (**b**)

An 88-year-old female patient with atrial fibrillation on coumadin presented to her physician with mental status changes for 1 week which were initially attributed to alprazolam. This continued despite withholding alprazolam and therefore outpatient routine head CT was ordered. The deep learning algorithm detected ICH and the study (representative image shown in Fig. 5a) was automatically reprioritized from routine to stat. As a result, the study was interpreted within 39 min to have acute intracerebral hemorrhage within the left anterior-medial temporal lobe (uncus). The patient was admitted and anticoagulation reversed with prothrombin complex concentrate and vitamin K, preventing further enlargement of the hemorrhage. Repeat CT of the head 36 h later showed stable hematoma without expansion, and a head CT 1 month later showed near complete resolution of the hemorrhage. The early diagnosis (39 min) likely led to critical early reversal of anticoagulation and stabilization of the hemorrhage instead of worsening, potentially lethal expansion in the setting of anticoagulation.

#### Case #2

A 76-year-old male who fell down a flight of stairs was treated non-operatively for a right scapula fracture, transverse process spinal fractures and a left flank hematoma. His initial head CT showed no acute findings, and he was discharged home after 2 days. The patient then presented for outpatient follow-up 6 weeks later with continued dizziness and bifrontal headaches, at which time a routine outpatient head CT was ordered. An acute/subacute left subdural hematoma was noted, and the study was automatically prioritized stat by the ICH detection algorithm. The study was read in 8 min and confirmed to show acute subdural hematoma (representative image shown in Fig. 5b). While the patient was initially managed with close observation non-operatively, he ultimately required emergent evacuation of acute on chronic subdural hemorrhages 12 days later at which time he had another head CT that was again identified by the algorithm as showing ICH.

### Neuroradiologist over-read results

Of the 34 presumed false positive studies from the clinical implementation phase where the algorithm detected ICH but the original dictating radiologist stated no hemorrhage, 4 of these were subsequently identified as showing probable hemorrhage with at least moderate confidence by the staff neuroradiologist over-reading the studies in blinded fashion without knowledge of the purpose of the study. Representative images and review of the cases are provided in the supplementary document (Figures S2 and S3).

## Discussion

We have shown that deep neural networks trained on large clinical imaging datasets can detect critical radiological conditions such as ICH with high accuracy (AUC of 0.846). Importantly, the proposed algorithm detected a variety of ICH cases from a highly heterogeneous dataset acquired clinically over a decade, without a priori information about the location of the hemorrhage and without controlling for factors such as scanner type, patient conditions or image acquisition parameters. The algorithm was then implemented into a live clinical radiology workflow optimization scenario where significant benefit on time to diagnosis of ICH in outpatients was demonstrated. To our knowledge, no other study has implemented a deep learning algorithm into clinical radiology workflow.

While head CT scans are often acted on by neurosurgeons in the acute setting concomitantly or even prior to radiologist interpretation, the outpatient setting is typically managed by family or internal medicine physicians who rely on a radiologist’s interpretation prior to acting. Our algorithm was therefore designed to operate in the outpatient setting, and our results demonstrated the clinical benefit of 96% faster diagnosis of 5 new ICH cases out of 347 routine head CTs (1.5%). Thus, the benefit of earlier diagnosis in the 1.5% of outpatients who may have an unknown ICH far outweighs the essentially negligible added burden of the algorithm on radiology worklist prioritization. Future studies may be able to utilize this now established workflow to also provide benefit in the inpatient or emergency setting, perhaps by identifying cases most likely to need neurosurgical intervention or by predicting appropriate intervals for follow-up head CT required to monitor progression of ICH. It is also important to note that these results were achieved within our integrated health system which has 24-h radiology coverage. In an outpatient radiology facility without 24-h radiology coverage, automated workflow optimization based on ICH detection will likely provide even more benefit.

Computer-aided diagnosis (CAD) has been an active area of research in the past five decades.^18^ Starting with detection of breast cancer on mammograms,^19^ CAD has been extended to several other diseases such as lung cancer,^20^ colon cancer^21^ and more recently several brain disorders such as Alzheimer’s disease.^22^ Despite all these efforts, only CAD for breast imaging has been widely adopted in clinical practice. However, even the effectiveness of that has been controversial with some studies supporting the benefits^23^ and others questioning the utility.^24^ Importantly, most clinical CAD systems are based on traditional computer vision techniques and do not utilize deep learning.

Deep learning methodology has proven to be effective in several domains, such as object detection, where it has outperformed traditional techniques as well as humans in computer vision competitions.^25^ In recent years, there has been increased interest in deep learning based CAD.^26^ For example, recently it was shown that accurate detection of diabetic retinopathy on retinal photographs is possible using a deep learning framework.^14^ Other applications of such frameworks include skin cancer detection,^15^ pulmonary nodule detection on CT images^16^ and cerebral microbleed detection on magnetic resonance images.^17^ One of the main advantages of deep learning based CAD over traditional CAD such as that used in mammography is that it automatically learns and extracts hierarchical levels of abstraction from raw images in a fully data-driven manner instead of using hand-crafted features. Typically, CAD systems provide a “second opinion” while the radiologist makes the final decision. In the proposed framework, the deep learning based ICH detection algorithm is working behind the scene and simply affects radiology read times for studies with detected ICH, however, future implementations could test its utility in other scenarios. For example, providing algorithm results to radiologists in realtime may have additional clinical benefit. This is supported by our data showing that approximately 10% of the initially “false positive” cases detected as having ICH by the algorithm but not by the original interpreting radiologist were determined to have probable ICH with at least moderate confidence during a blinded over-read. Whether these subtle, overlooked hemorrhages will ultimately prove to be clinically actionable remains a subject of future work that will require a larger prospective study.

Despite the promising results, several limitations deserve comment. First, radiological reports were used as the reference to extract the label for each head CT, and the accuracy of this diagnosis is unknown. The possible errors introduced may have reduced the accuracy of the predictive model. The potential remedy for this limitation would be large numbers of over-reads by radiologists, possibly via crowd-sourcing.

In its current form, the algorithm does not specify the location of the ICH which is detected. This would be a helpful feature to assist the radiologist in efficient evaluation of the results, particularly in cases where the radiologist does not see anything yet the algorithm detects an abnormality. While CNNs are capable of extracting features relevant to the ICH detection task in a fully automated manner, those features are not necessarily understandable to humans in their native form. Importantly, there has been significant effort in this direction in recent years in the computer vision community. Two promising approaches are “Region-based Convolutional Neural Network” (R-CNN)^27^ and “Gradient-weighted Class Activation Mapping” (GRAD-CAM).^28^ R-CNN combines region proposals with features computed by the CNN while GRAD-CAM uses the gradients of the target in order to produce a localization map highlighting the important regions of the image that contributed the most to the prediction. However, both R-CNN and GRAD-CAM currently work on 2D images and are not necessarily straightforward to apply to the 3D images used in the current study.

Other than a reduction in time to diagnosis, patient outcomes were not formally evaluated in the current study. Indeed, a prospective study to look at outcomes would likely need to operate for several years to measure benefit. However, we did provide detailed anecdotal evidence of clinical benefit in 2 out of the 5 new cases of ICH that were identified on the outpatient head CT list. Moreover, time to communication of critical findings such as ICH is tracked by nearly every radiology department in the US, and the algorithm showed clear benefits on this metric. Finally, time to diagnosis is important to patients and their families no matter the clinical outcome. This work therefore lays the foundation for future, large prospective studies of patient outcomes, perhaps after randomization to using or not using the algorithm.

We fed the network approximately 37,000 three-dimensional 3D head CT studies (each study included all 2D image slices from the 3D study). Given that we used 3D architecture, the presented network was the largest that could fit within the memory of the GPUs we used for this study. More complicated models may increase the performance, however, would require parallelizing the model across more GPUs, which we did not have access to for the current study. Moreover, downsampling the images slightly to 256 × 256 × 24 was also required due to hardware limitations, and future studies may be able to slightly improve performance by using additional slices and larger (native) matrix sizes.

## Methods

### Imaging dataset

46,583 non-contrast head CT studies from 31,256 unique adult patients were collected retrospectively from our integrated healthcare system’s picture archiving and communication system. None of these head CT studies had been previously used for research or published, and they were not taken from publically available databases. Studies were required to have an associated complete clinical dictation report along with at least 20 axial 2D slices. The Geisinger institutional review board reviewed the study protocol and determined the work to be exempt. The head CT studies were acquired using 17 scanners from 4 different manufacturers located in facilities across our health system in Pennsylvania from 2007–2017. Each study contained a variable number of 2D axial images (20–378) with slice thicknesses ranging from 0.625–5.0 mm. Studies were not controlled for scanning methodology or settings such as pixel spacing, scan time and radiation dose. The dataset was randomly divided into training (37,084 studies) and testing (9499 studies) sets. During a 3-month implementation phase, the algorithm processed 347 studies for automatic real-time radiology worklist queue re-prioritization (called production data hereafter). The percentage of head CT studies derived from the inpatient, outpatient and emergency settings was 20, 34, and 46%, respectively, for both the training and testing datasets. Of the 37,084 studies in the training set, 26.8% were labeled as having ICH. The characteristics of the dataset are summarized in Table 1.

**Table 1:** Baseline characteristics of the data set

	Training	Testing	Production
Number of radiology facilities	13	13	13
Number of unique individuals	24,882	6,374	329
Number of studies	37,084	9,499	347
Number of 2D images	1,624,068	331,092	12,532
Collection Period	2007–2017	2007–2017	01/06/2017–03/24/2017
Slice thickness range (mm)	0.625–5		
Number of slices per study (range)	20–378		
2D image resolution (pixel)	512 × 512		
Age, mean (SD) years	58.89 (19.36)	58.87 (19.37)	61.36 (20.11)
Gender (% female)	47.02 %	46.98 %	45.90 %
Patient Type	Outpatient, Inpatient and Emergency	Outpatient, Inpatient and Emergency	Outpatient and Inpatient
Study Critical Level	Routine and Stat	Routine and Stat	Routine

### Labels

The binary label for each imaging study (negative or positive ICH) was inferred from the official clinical radiology reports associated with the study (generated by an interpreting attending staff radiologist) as detailed in the supplementary material. Note that the labels were assigned in a binary fashion to the entire head CT imaging study, not single images from each head CT.

### Preprocessing of imaging data

Each head CT study contained a variable number of 512 × 512 (pixel) axial images mainly due to head size differences and variable slice thicknesses. This was standardized by resampling each study to 24 image slices using a standard cubic spline interpolation. Each slice was also resized to 256 × 256 using a cubic spline interpolation. These two steps helped each study to achieve a uniform dimensionality of 24 × 256 × 256 regardless of the slice thickness. In CT imaging, the amount of X-ray radiation absorbed by tissues at each location in the body is mapped to Hounsfield units (HU). Water is always set to 0 HU, while air is −1000HU, and bones have values between several hundred to several thousand HU. To increase the contrast of the images, a “blood window” was applied to each study (window level:40, window width:80). Finally, to increase the training data and make it more balanced, each study was augmented by applying random horizontal and vertical translation (±0–20 pixels), rotation (±0–15°) and mirroring (horizontal) to generate new augmented studies from each original study in the training dataset (20 and 80 augmented studies from each negative and positive study, respectively).

### Algorithm development

In recent years, deep CNN have proven effective in a variety of vision tasks such as object detection^29^ and image segmentation.^30^ Deep learning is a data-driven methodology for training large neural networks to map the input data (in this case, head CT images) to the desired outputs (in this case, negative or positive ICH) through a nonlinear mathematical function. Training such networks requires large amounts of imaging data for which the ground truth labels are known. By feeding the pixel intensities of each image and the associated label into the deep learning network during the training phase, network parameters change so that more accurate prediction can be made by reducing the error (or loss) resulting from comparing the generated output to the ground truth labels. This process is repeated many times for each imaging study until the network is trained. A trained network should be able to make the desired prediction on unseen “test” data (and not just on the data it was trained on).

The architecture we used in this study is illustrated in Fig. 1. This architecture has five convolutional layers and two fully connected layers (aside from the max pooling and normalization layers). The convolutional network was initialized randomly from a normal distribution centered at zero. Stochastic gradient descent was used to train the proposed network. The whole dataset was randomly divided into three parts for training (~75%), cross validation (~5%) and testing (~20%). The performance of the algorithm was measured using the AUC. After every epoch training phase, the AUC was measured on the cross validation dataset and the network parameters (weights of the model) were saved. The algorithm was trained until near zero loss was obtained on the training dataset (overtraining). The parameters at the point corresponding to the highest AUC on the cross validation dataset were chosen for the final trained algorithm. The generalization performance of the network was measured using the unseen testing dataset. To improve the accuracy and robustness of the algorithm, an ensemble of four networks were trained on the same dataset. The final prediction was made by thresholding the average over the predictions of the ensemble. We used the Caffe deep learning package^31^ to train our model.

### Clinical implementation

The algorithm was implemented as a quality improvement tool for radiology interpretation workflow optimization. At our institution, similar to most institutions, all radiological exams are either ordered as “stat” or “routine”. All patients in the emergency department setting are designated “stat” by default. All other inpatient or outpatient studies are designated as “stat” or “routine” by the ordering provider in the electronic medical record system. This decision is most often based on the clinical status of the patient and pretest probability of a critical finding; both of which are usually determined by the referring physician. The highest critical level is called “stat,” and exams with this status are prioritized in the radiology worklist queue. The reading list is sorted by a ruled-based engine with the stat exams on the top, sorted by the time they were performed. Radiologists interpret the exams starting from the top of the reading list.

To implement the algorithm, a data pipeline was built to transfer head CT studies (in near real time) for every routine non-contrast head CT study across the entire health system to the computational server containing the trained algorithm. The algorithm then processed the exam and generated a binary output (negative or positive ICH). If the results were positive, the priority of the study was upgraded to “stat” and the reading list was updated in real-time. If the results were negative, the priority of the study was not changed. The interpreting radiologists were not aware of the re-prioritization process. Figure 4 illustrates the clinical implementation of the ICH detection algorithm at our institution.

### Neuroradiologist over-read

To determine whether the algorithm correctly identified any subtle ICH which were potentially not seen by the original dictating radiologist, an attending staff neuroradiologist blinded to the study reviewed all false positive studies (from the production dataset) and was asked to state whether there was or was not ICH present. This neuroradiologist was allowed to examine both prior and future studies in blinded fashion, and was not able to see the clinical history or dictated reports in order to make this determination.

### Statistical analysis

The 95% confidence intervals for sensitivity and specificity of the algorithm at the operating point were computed using exact binomial proportion confidence intervals.^32^ The 95% confidence interval of the AUC was calculated based on the Delong method which is asymptotically exact.^33^ The *p*-value for comparing median read-times was calculated empirically via permutation test repeated one million times.

### Data availability

The datasets generated for the current study are available on reasonable request by contacting the corresponding author. The Caffe deep learning package is open source.^31^

## Electronic supplementary material

Supplementary Material

## Electronic supplementary material

**Supplementary information** accompanies the paper on the *npj Digital Medicine* website (10.1038/s41746-017-0015-z).
