# Supplementary material for: Advanced machine learning in action: identification of intracranial hemorrhage on computed tomography scans of the head with clinical workflow integration
Source: NPJ Digit Med. 2018 Apr 4;1:9. doi: 10.1038/s41746-017-0015-z (PMC6550144; doi:10.1038/s41746-017-0015-z)
Supplement: Supplementary file 1 — Supplementary Material [file 41746_2017_15_MOESM1_ESM.docx]

# Supplementary Information

# Labeling of Radiological Reports:

The binary label of each report (negative or positive ICH) was inferred by trained research assistants under the supervision of an experienced neuroradiologist for 25% of the data. A study was labeled positive if any type of intra-axial or extra-axial hemorrhage was identified in the report, regardless of size or acuity. The rest of the labels were derived automatically using the Apache cTAKES natural language processing package as described below.

##

## Automatic Labeling of Radiological reports:

Using the Radiology Information System (RIS), head CTs were identified and all radiological reports were extracted. The reports were then parsed using regular expressions to extract only the impression and/or findings. This approach was used to help reduce the amount of annotations and possible noise that could be introduced when running natural language processing. Once the reports were pre-processed, Apache cTakes (1) was used to extract annotations from the reports.

The cTakes pipeline and models used were mainly out of the box functionality from the clinical pipeline provided by the software using the Unified Medical Language System (UMLS) release 2015AB as the terminology dictionary. This included the normal processing of sentences and words to apply additional advanced pipeline features. One of the most important features was using the polarity module, so we could identify negation in terms. In addition to the above processing, we added a brute force method for labeling the anatomical location of annotations when available. Also, we added a custom writer and limited the output to document ID, UMLS CUI, CUI description, the raw annotated text, start position, stop position, negation (Boolean), generic use of annotation (Boolean) and uncertainty of annotation (Boolean). If the concept code #1386000 (Intracranial hemorrhage (disorder)) was present for a given report, then it was automatically labeled as “ICH positive”. Otherwise, it was labeled as “ICH negative”. The results were loaded to a SQL Server where additional processing or additional UMLS information could be added as needed.

# Examples of False Positives of the ICH Detection Algorithm:

Figure S1 shows five representative examples of false positive cases including arterial calcification, global cerebral volume loss and ischemic changes.

# Clinical Implementation Results:

The confusion matrix of the 347 non-stat head CT studies that were processed using the proposed algorithm during the clinical implementation phase is shown in Table S1.

# Neuroradiologist Over-read Results:

Of the 34 false positive studies that were dictated by the original interpreting radiologist as having no ICH and subsequently over-read by a blinded neuroradiologist, four were found to have ICH with moderate confidence. Figure S2 illustrates the representative slices and the over-reader comments for three of those cases. The fourth case is illustrated in Figure S3.

# Supplementary References:

1. Savova GK, Masanz JJ, Ogren P V, Zheng J, Sohn S, Kipper-Schuler KC, et al. Mayo clinical Text Analysis and Knowledge Extraction System (cTAKES): architecture, component evaluation and applications. J Am Med Informatics Assoc. The Oxford University Press; 2010;17(5):507–13.

**Table S1: Confusion matrix of production data**

|  | | **Ground truth (from the radiologist report)** | |
| --- | --- | --- | --- |
|  |  | Positive ICH | Negative ICH |
| **Algorithm prediction** | Positive ICH | 60 | 34 |
|  | Negative ICH | 26 | 230 |

| 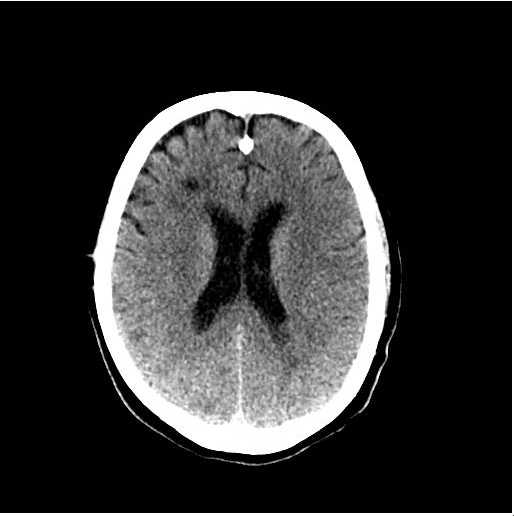 | 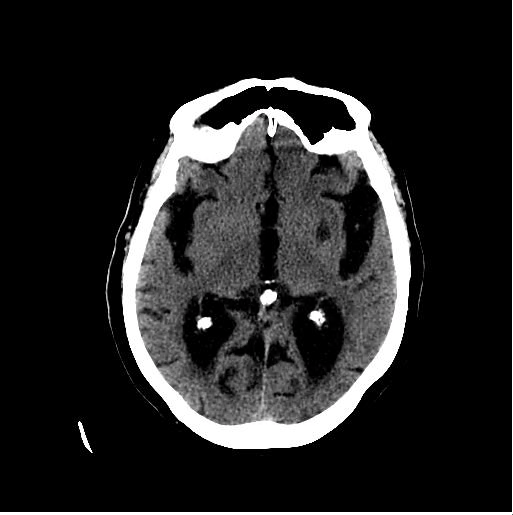 | 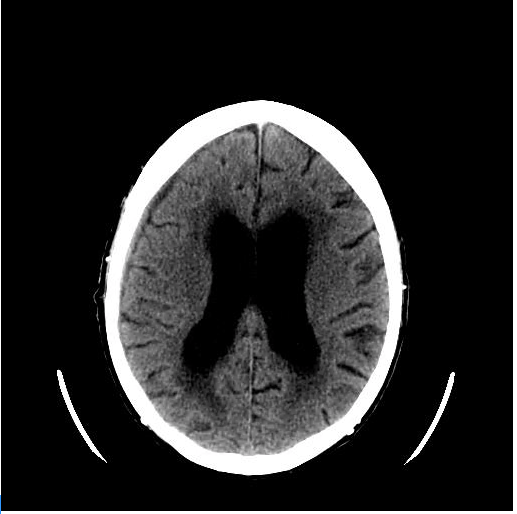 | 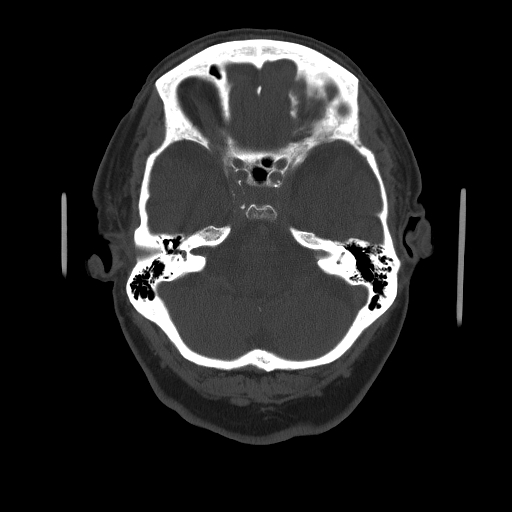 |
| --- | --- | --- | --- |
| **Patient Status:** Outpatient  **Finding:** Acute and subacute ischemic infarcts scattered throughout the bilateral cerebral hemispheres | **Patient Status:** Outpatient  **Finding:** Global cerebral volume loss | **Patient Status:** Outpatient  **Finding:** hydrocephalus | **Patient Status:** Outpatient  **Finding:** Intracranial arterial calcifications |
| **Figure S1:** Examples of algorithm errors (false positives) | | | |

| 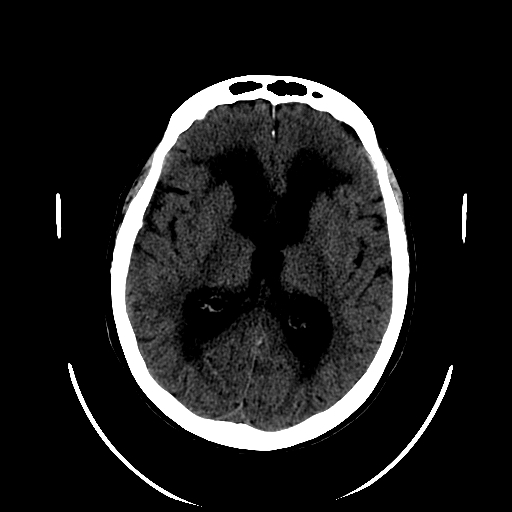 | 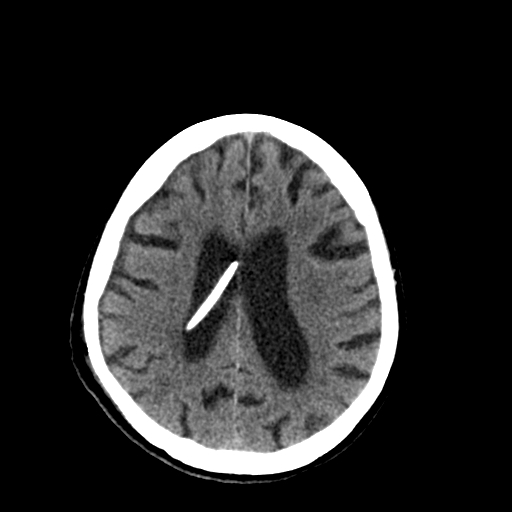 | 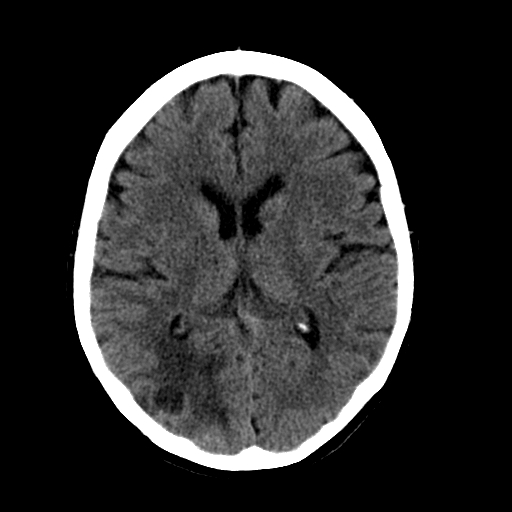 |
| --- | --- | --- |
| Over-reader impression: Tiny left frontal subdural hemorrhage which resolved on subsequent imaging. The patient has a history of subarachnoid hemorrhage and coiled aneurysm and was being scanned for presumed worsening of communicating hydrocephalus. | Over-reader impression: Female with history of normal pressure hydrocephalus who underwent VP shunt catheter placement. Her post-operative CT showed a thin subdural hemorrhage that was not identified prospectively by the original interpreting radiologist. | Over-reader impression: Female with lung cancer metastatic to the brain. Micro hemorrhages were likely present within the lesions as shown. |
| **Figure S2:** The representative image along with the over-reader comments for three cases | | |

| 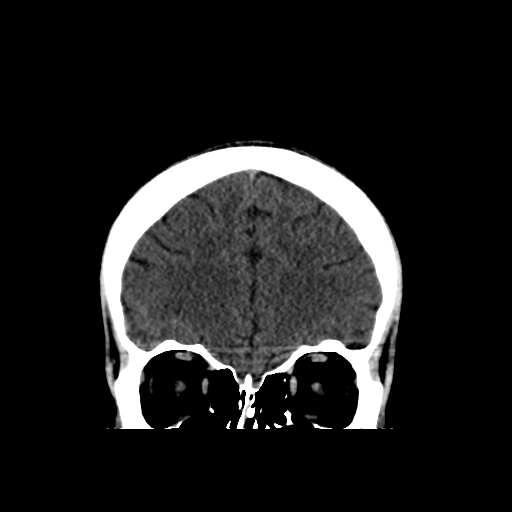 | 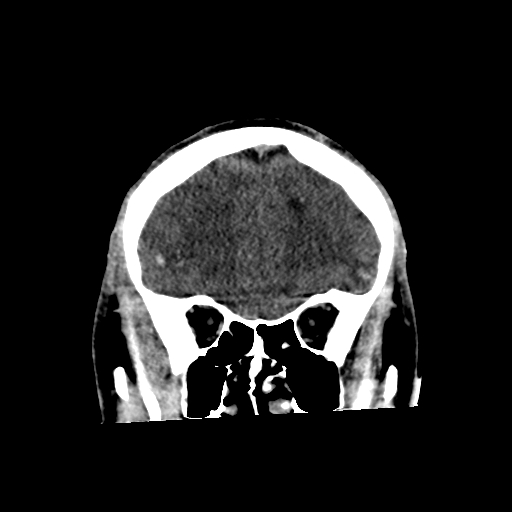 | 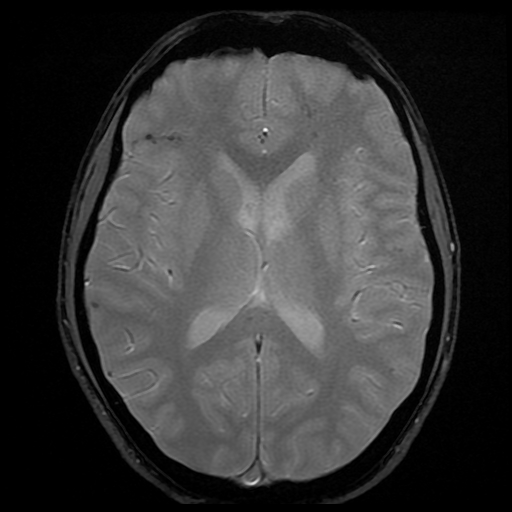 |
| --- | --- | --- |
| Current Study | Prior Study | MRI |
| **Figure S3:** Male in head-on motor vehicle collision with parenchymal hemorrhagic contusions that mostly resolved on the CT of interest. Over-reader believed there was likely resolving ICH and/or encephalomalacia. The prior as well as MRI of the brain (showing susceptibility on GRE consistent with hemorrhage) are provided to better depict the subtle ICH. | | |


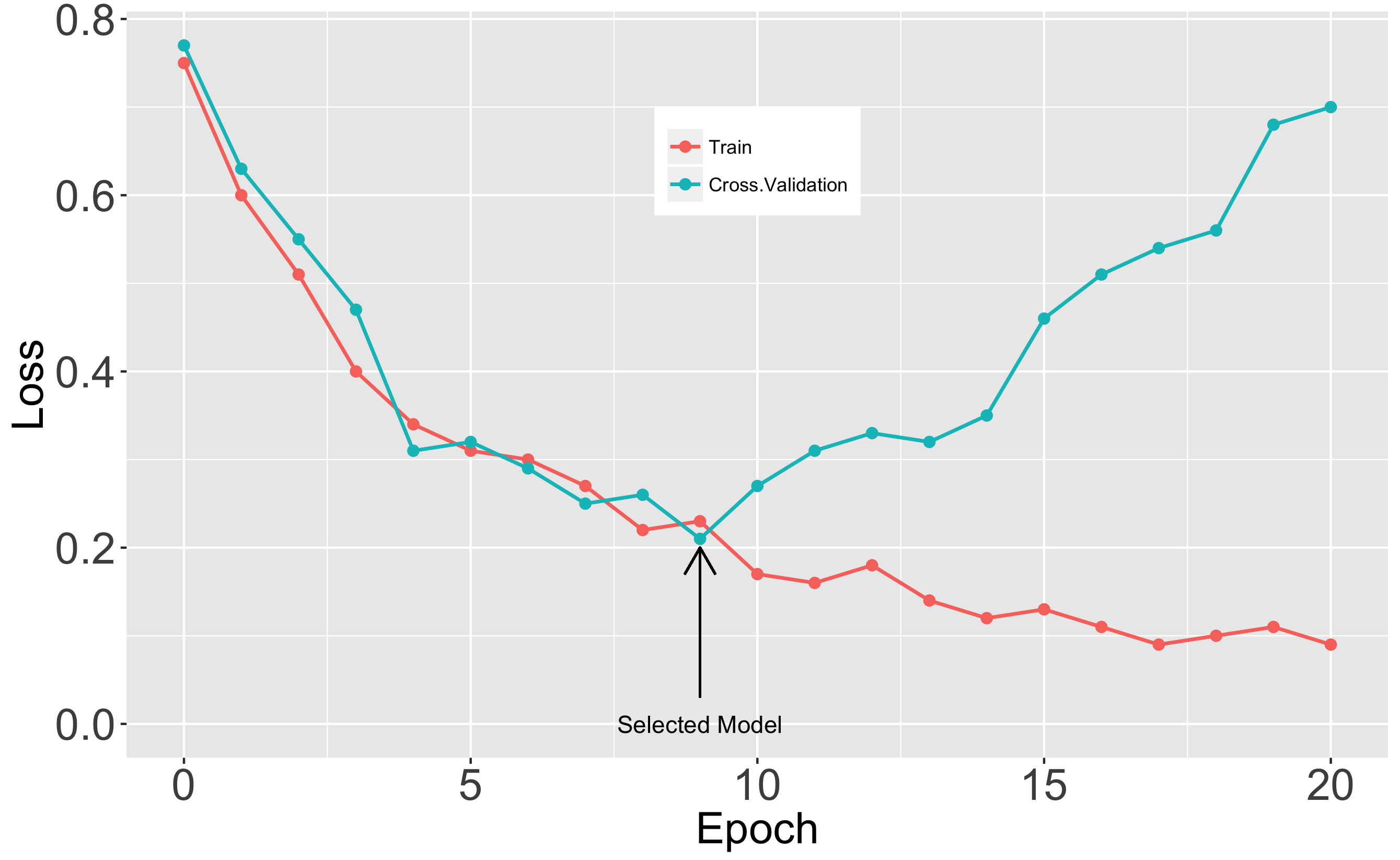


**Figure S4:** Train and cross validation loss during CNN model training. The minimum cross validation loss was reached at epoch #9 at which point the corresponding model was selected for testing.
